# Noise-Resilient Bioacoustics Feature Extraction Methods and Their Implications on Audio Classification Performance: Systematic Review

**DOI:** 10.2196/80089

**Published:** 2025-12-16

**Authors:** Geofrey Owino, Bernard Shibwabo

**Affiliations:** 1 School of Computing and Engineering Sciences, Strathmore University, P.O. Box 75584, Nairobi, 00200, Kenya, 254 721913968

**Keywords:** bioacoustics classification, noise robustness, feature extraction, denoising techniques, audio signal processing, machine learning, deep learning, real-world deployment

## Abstract

**Background:**

Bioacoustics classification plays a crucial role in ecological surveillance and neonatal health monitoring. Infant cry analysis can aid early health diagnostics, while ecological acoustics informs conservation. However, the presence of environmental noise, signal variability, and limited annotated datasets often hinders model reliability and deployment. Robust feature extraction and denoising techniques have become critical for improving model robustness, enabling more accurate interpretation of acoustic events across diverse bioacoustic domains under real-world conditions.

**Objective:**

This review systematically evaluates advancements in noise-resilient feature extraction and denoising techniques for bioacoustics classification. Specifically, it explores methodological trends, model types, cross-domain transferability between clinical and ecological applications, and evidence for real-world deployment.

**Methods:**

A systematic review was conducted by searching 8 electronic databases, including IEEE Xplore, ScienceDirect, Web of Science, ACM Digital Library, and Scopus, through December 2024. Eligible studies entailed audio-based classification models and applied empirical or computational evaluations of bioacoustics classification using machine learning or deep learning methods. In addition, studies also included explicit or implicit consideration of noise. Two reviewers independently screened studies, extracted data, and assessed quality. Risk of bias was assessed using a customized tool, and reporting quality was evaluated using the TRIPOD (Transparent Reporting of a Multivariable Prediction Model for Individual Prognosis or Diagnosis) checklist.

**Results:**

Of the 5462 records, 132 studies met the eligibility criteria. The majority (112/132, 84.8%) of studies focused on model innovation, with deep learning and hybrid approaches being the most dominant. Feature extraction played a critical role, with 96.2% (127/132) of studies clearly demonstrating feature extraction. Mel frequency cepstral coefficients, spectrograms, and filter bank-based representations were the most common feature representations. Nearly half (62/132, 47%) of the studies incorporated noise-resilient methods, such as adaptive deep models, wavelet transforms, and spectral filtering. However, only 14.4% (19/132) demonstrated real-world deployment across neonatal care and ecological field settings.

**Conclusions:**

The integration of noise-resilient techniques has significantly improved classification performance, but real-world deployment and proper use of denoising strategies in various datasets remain limited. Cross-domain synthesis reveals shared challenges, including dataset heterogeneity, inconsistent reporting, and reliance on synthetic noise. Future work should prioritize harmonized benchmarks, cross-domain generalization, and deployment, as well as opportunities for transferability.

## Introduction

### Background

Bioacoustics, the study of sound produced by biological organisms, has become an essential tool for understanding ecological dynamics, monitoring biodiversity, and health diagnostics and monitoring [1]. Bioacoustics signals, for instance, birdcalls, marine mammal sounds, human sounds, and infant cries, provide information about species behavior, ecosystem health, and human well-being [2]. In neonatal care, infant cry analysis is explored as a noninvasive marker of health and a potential tool for early diagnostics and caregiver decision support. In ecological monitoring, passive acoustic sensors are increasingly deployed for biodiversity surveillance, species identification, and environmental assessment. Passive acoustic monitoring has been significant in tracking population dynamics and detecting anomalies in biological sound patterns [3].

Bioacoustics signals are also used in health care as noninvasive markers for diagnosing respiratory conditions, neurological disorders, and infections such as sepsis [4]. These signals are increasingly becoming central to digital health. Infant cry analysis is one of the emerging core areas in digital health. It is a practical avenue for early risk triage, remote monitoring, and real-time decision support in neonatal care [5]. Other pathological audio domains, such as lung sound classification for respiratory disease diagnosis, have also been systematically reviewed [6-8]. These reviews reinforce the importance of robust audio pipelines in clinical monitoring. Similarly, acoustic monitoring is crucial for species identification and biodiversity assessments, particularly in remote or inaccessible regions, and is focal to wildlife conservation [9]. Despite rapid progress, both clinical and ecological bioacoustics applications are constrained by one fundamental limitation, noise interference, which undermines the reliability and interpretability of classification models in real-world deployments.

The most persistent challenge in bioacoustics analysis is environmental noise contamination, which degrades signal quality and reduces classification accuracy. Noise arising from human activity, equipment artifacts, and overlapping acoustic sources complicates the extraction of meaningful features. Clinical environments are also acoustically challenged by alarms, caregiver speech, ventilation, and room reverberation. These factors reduce signal quality, thereby limiting the effectiveness of machine learning-based audio classification models [10]. Feature extraction forms the critical interface between raw bioacoustics waveforms and downstream classifiers. While traditional feature extraction techniques remain fundamental in audio classification, they exhibit high noise sensitivity, leading to feature distortion and reduced classification accuracy [11]. Numerous noise-resilient techniques such as wavelet filtering, adaptive spectral subtraction, and hybrid deep neural embeddings have been proposed to tackle these challenges. However, their evaluation remains fragmented and inconsistent across domains. No consensus exists regarding the most effective denoising or feature extraction strategies for bioacoustic classification, nor how these choices influence model deployment or interpretability under realistic noise conditions [10,12].

Persistent research gaps remain in evaluating the effectiveness and generalizability of noise-resilient feature extraction methods across domains. Many studies rely on controlled or synthetic noise settings, limiting ecological and clinical applicability. Benchmark initiatives such as Stowell’s roadmap and the BirdSet dataset have advanced standardization in ecoacoustics but do not yet address cross-domain noise resilience or deployment metrics. Reporting of noise protocols and preprocessing remains inconsistent, and evidence of real-world deployment—especially in neonatal and field settings—is scarce. To bridge these gaps, this systematic review aims to (1) map methodological trends in noise-resilient feature extraction and denoising; (2) quantitatively evaluate their impact on classification performance under varying noise conditions; (3) examine evidence for real-world deployment and cross-domain generalization; and (4) identify limitations and future research priorities to advance robust, interpretable, and deployable bioacoustic systems.

Persistent research gaps remain in evaluating the effectiveness of noise-resilient feature extraction methods across different bioacoustics applications [13,14]. Many studies assess models in controlled or synthetic noise conditions, limiting ecological and clinical applicability as models fail to reflect the complexity of real-world acoustic environments [15]. Benchmark initiatives such as Stowell’s roadmap explicitly call for community standards and comparable benchmarks in bioacoustics deep learning [13]. “The Benchmark of Animal Sounds,” proposed by Hagiwara and colleagues to standardize evaluation across multiple animal-sound datasets [14], and a large-scale dataset for audio classification in avian bioacoustics, “BirdSet,” were also created to address dataset fragmentation in avian tasks [16]. However, these efforts remain largely species-specific with no noise protocols, denoising baselines, or clinical (neonatal intensive care unit [NICU]) deployment metrics, underscoring the need for a minimal evaluation to enhance transition to deployment. Existing reviews largely focus on ecoacoustic pipelines and tasks rather than cross-domain noise robustness or deployment in clinical settings [17,18].

### Related Work

In addition, reporting of noise protocols and preprocessing is inconsistent, limiting comparability in domains. Evidence on deployment is scarce, with only a minority of studies tested in neonatal or ecological field settings. Finally, little cross-domain synthesis exists to establish whether techniques effective in infant cry analysis generalize to ecological monitoring, and vice versa. To address these gaps, this systematic review focused on four objectives: (1) mapping methodological trends in feature extraction, denoising, and model development; (2) evaluating classification performance under noisy conditions; (3) assessing evidence for deployment and cross-domain transferability; and (4) synthesizing limitations and future priorities to guide the development of robust, scalable bioacoustics systems.

Bioacoustic recordings across domains are degraded by environmental and clinical noise, limiting the reliability of feature extraction and classification techniques [19]. Noise interference remains a major obstacle in bioacoustics research, stemming from natural background sounds, overlapping vocalizations, human-induced disturbances, and equipment-related artifacts [20]. Low signal-to-noise ratios (SNRs) degrade the clarity of acoustic signals, making it difficult to extract meaningful features [21]. In urban environments, background noise from traffic, industrial activity, and human movement significantly reduces the accuracy of automated species identification. Similarly, in neonatal health care settings, excessive ambient noise negatively affects infant cry-based medical diagnostics, leading to misclassification and reduced sensitivity [22]. This section summarizes literature on feature extraction and denoising techniques to benchmark the gaps for data synthesis.

Feature extraction is a vital phase in bioacoustics classification; it transforms signals into meaningful representations for machine learning and deep learning models. Traditional methods such as Mel frequency cepstral coefficients (MFCCs), spectrograms, and linear predictive cepstral coefficients (LPCCs) have been widely used due to their effectiveness in capturing essential acoustic properties. MFCCs, in particular, have been extensively applied in speech and sound classification tasks due to their ability to model human auditory perception [23]. Spectrogram-based methods provide time-frequency representations, enabling the visualization and analysis of complex vocalization patterns [24]. LPCCs have been used in general acoustics research for feature extraction due to their capacity to model the vocal tract system in speech signals [25]. However, these feature techniques perform well in controlled environments but struggle with real-world noise.

High classification error rates arise when extracted features are distorted by background interference, reverberation, and overlapping signals [10]. These limitations necessitate continuous development of advanced noise-resilient feature extraction techniques as bioacoustics moves toward more complex field applications. Research is moving toward noise-resilient feature extraction methods that integrate signal processing, machine learning, and deep learning–based methods. These methods are objective in feature robustness enhancement, mitigation of noise artifacts, and improving classification accuracy in dynamic environments.

Denoising techniques have been used before feature extraction to enhance signal quality and after feature extraction to enhance model performance. Several techniques have been used extensively; among them, spectral subtraction, Wiener filtering, and wavelet-based denoising are used extensively. Spectral subtraction reduces stationary background noise by estimating the noise spectrum during nonvocalization periods and subtracting it from the noisy signal [26]. However, spectral subtraction can introduce artifacts such as musical noise, which may distort classification results, making it less effective for nonstationary noise [27]. Wiener filtering reduces the mean square error between the estimated clean signal and the observed noisy input, adapting to local SNRs [28]. It has been used successfully in bioacoustics monitoring and medical diagnostics, where background noise levels vary dynamically [4].

Wavelet-based denoising uses wavelet transforms to decompose data into distinct frequency bands. This technique reduces high-frequency noise while maintaining salient biological acoustic properties by selectively attenuating noise components at particular scales [21]. Marine bioacoustics has effectively used wavelet denoising to enhance the detection of low-frequency vocalizations, such as whale sounds, in noisy underwater environments [29]. Adaptive filtering dynamically adjusts its parameters in response to changing noise conditions, making it particularly effective for field-based bioacoustics monitoring [30]. Adaptive filtering has been used in avian bioacoustics, where real-time adjustments help maintain signal clarity despite weather fluctuations and overlapping birdcalls [3].

Advanced neural network architectures have shown significant improvements over conventional techniques for managing noisy bioacoustics data. Recurrent neural networks (RNNs) and convolutional neural networks (CNNs) have been essential in this development. RNNs are well suited to modelling time-based relationships in sequential data, and CNNs excel at extracting spatial characteristics from spectrogram representations of audio signals. These networks improve classification accuracy in diverse acoustic situations by learning noise-invariant representations [31,32]. Hybrid models and their variants have improved classification accuracy in diverse noisy environments. Convolutional recurrent neural networks (CRNNs) combine the advantages of RNNs and CNNs by integrating temporal sequence modeling and spatial feature extraction, enabling CRNNs to efficiently identify intricate patterns in bioacoustics data, even in noisy environments [33].

Generative adversarial networks (GANs) have been used to improve model robustness by generating synthetic training data that simulate real-world noise conditions [34]. GANs allow models to learn from an additional, diverse set of scenarios, refining their generalization capabilities. Additionally, training datasets have been expanded through data augmentation techniques and contextual noise to improve classification performance [35]. Finally, incorporating noise-adaptive attention mechanisms into audio classification models allows selective focus on signal components that are less affected by noise, thereby enhancing classification performance. While these approaches often improve accuracy under noise, latency demands can hinder on-device or field deployment without model compression or edge-aware design.

Evaluation protocols vary widely, with some studies using synthetic overlays with fixed SNR grids while others use in situ recordings with uncontrolled noise. The evaluation metrics and reporting details differ substantially. Underreporting of noise types and inconsistent disclosure of denoising complicate cross-study comparisons and can inflate perceived robustness. Community efforts such as multidataset animal-sound benchmarks and large avian corpora have improved scale and comparability but rarely prescribe explicit noise protocols or denoising baselines [14,16]. Furthermore, there are no NICU-specific deployment metrics. These gaps motivate a tiered evidence strategy, core noise-resilient versus comparator pipelines, and a structured synthesis.

This systematic review aims to summarize existing literature, pinpoint performance patterns, and draw attention to research gaps in the development of classification models for noise-resilient bioacoustics. In order to guide future research toward more scalable, generalizable, and noise-resilient bioacoustics systems, this study attempts to address these issues and offer an organized overview of the topic. Furthermore, we synthesize the effect direction, transferability, and deployment evidence across infant-cry and ecological settings.

### Objectives

The core objective of this study is to systematically review and synthesize advancements in noise-resilient bioacoustics feature extraction methods, evaluating their implications on audio classification performance in real-world noise. Specifically, we (1) map methodological trends (features, denoisers, models, and study designs); (2) quantify performance under noisy conditions relative to clean baselines; (3) assess cross-domain transferability and evidence of deployment (clinical, field, or edge); and (4) identify limitations and priorities to guide future research and implementation of robust, scalable bioacoustics classification systems.

To operationalize this objective aim, the review pursued four specific objectives: (1) identify methodological trends in feature extraction, denoising, and machine learning models applied to bioacoustics classification under noise; (2) evaluate performance outcomes reported across studies, including accuracy, precision, recall, and *F*_1_-score, with attention to differences between clinical and ecological domains; (3) assess deployment evidence by analyzing whether and how methods have been tested or implemented in real-world conditions, and to what extent they demonstrate cross-domain robustness; and (4) synthesize limitations and future priorities, highlighting dataset challenges, methodological gaps, and opportunities for advancing noise-resilient bioacoustics analysis.

By integrating findings from multiple studies, the review seeks to provide practical recommendations for both academic research and real-world implementations, ensuring the development of more robust, scalable, and adaptive bioacoustics classification systems. Based on these objectives, the following review questions (RQs) were formulated to align closely with the study’s scope and focus:

- RQ 1.1: What feature extraction, denoising or enhancement, and machine learning model approaches are used to achieve noise-resilient bioacoustics classification? This question synthesizes traditional signal-processing methods (eg, MFCC, LPCC, and per-channel energy normalization [PCEN]), denoisers (eg, spectral subtraction, Wiener, wavelet, and deep denoisers), and model classes (eg, CNN, RNN, CRNN, and transformers) and documents prevailing study designs.
- RQ 1.2: How do these pipelines perform under noisy conditions compared with clean baselines, and what metrics and noise protocols are reported? This question extracts accuracy, precision, recall, and *F*_1_-score (and area under the receiver operating characteristic curve [AUC] where available); summarizes effect direction (Δ vs clean); and notes noise protocol transparency (type, SNR grids, and synthetic vs in situ).
- RQ 1.3: To what extent have these methods been deployed or prospectively evaluated in real-world settings, and how transferable are they across clinical (infant-cry) and ecological (wildlife) domains? This question examines model evaluation in ward, field, and edge environments; considers scalability and latency constraints; and assesses cross-domain robustness and generalizability.
- RQ 1.4: What limitations and risks of bias recur across studies, and what priorities should guide future work? This question identifies dataset imbalance, synthetic-only noise, reporting gaps (noise type, SNR, and denoising details), and distills priorities such as standardized noise protocols, benchmark design, and real-time or self- or federated-learning approaches.

## Methods

### Methodological Approach

This study follows a systematic review approach to analyze advancements in noise-resilient bioacoustics feature extraction methods and their implications on audio classification performance. To ensure transparency, reproducibility, and methodological rigor, this review followed the PRISMA (Preferred Reporting Items for Systematic Reviews and Meta-Analyses) 2020 guidelines for systematic reporting [36,37]. The Methodological Expectations of Cochrane Intervention Reviews (MECIR) standards were also used for study selection and evaluation [38]. The search and analysis were tailored using the PICO (population, intervention, comparison, outcome) framework to focus on studies relevant to the review objectives.

### Search Strategy

#### Information Sources

A comprehensive search was executed across 8 electronic databases—IEEE Xplore, ScienceDirect, Google Scholar, Web of Science Core Collection, ACM Digital Library, Scientific Electronic Library Online, China National Knowledge Infrastructure, and Scopus—yielding 5462 records. The search targeted peer-reviewed journal and conference papers published through 2024, in English and selected non-English (Spanish, Portuguese, Chinese, and French) languages. The search terms were developed based on the PICO framework in Multimedia Appendix 1, ensuring precision and relevance to the study’s scope.

#### Population (P)

Terms targeting bioacoustics audio data, such as “bioacoustics,” “animal vocalizations,” “bird calls,” “marine mammal sounds,” “infant cries,” and "biological acoustic signals.”

#### Intervention (I)

Keywords related to noise-resilient feature extraction methods, including “noise-resilient feature extraction,” “denoising techniques,” “MFCC,” “spectrogram,” “convolutional neural networks (CNNs),” “recurrent neural networks (RNNs),” “hybrid models,” and “attention mechanisms.”

#### Comparison (C)

Keywords related to evaluating the performance of different noise-handling techniques, such as “spectral subtraction,” “adaptive filtering,” “augmentation,” and “attention mechanisms,” against baseline approaches.

#### Outcome (O)

Keywords emphasizing classification performance and robustness, such as “classification accuracy,” “precision and recall,” “robustness to noise,” “scalability,” and “real-world applications.”

To cater to the non-English studies, the search terms were inadvertently translated into each target language, combined with controlled‐vocabulary headings where available. This multilingual strategy ensured maximal coverage of relevant noise-resilient bioacoustics classification studies. In addition to translation, filters were set to yield non-English relevant languages in the specific languages. Boolean operators (AND, OR) were used to combine and refine terms, and search strings were adapted for each database. For instance, the search query for Google Scholar was “bioacoustics” OR “infant cry classification” AND “animal vocalization recognition” AND “feature extraction” AND (“MFCC” OR “spectrogram” OR “wavelet”) AND “classification model” AND (“denoising” OR “noise robust” OR “signal enhancement”). The full search query syntax used for each database is provided in Multimedia Appendix 2.

### Eligibility Criteria

Inclusion and exclusion criteria were clearly defined to ensure methodological consistency and relevance to the objectives of this review. Studies were considered eligible for inclusion in the review if they involved the classification of bioacoustics signals, such as those from animals, birds, marine mammals, or human infants, using feature extraction methods or denoising techniques in real-world or noisy environments.

To preserve both comprehensiveness and focus, we defined 2 tiers of evidence. Tier A entails all noise-resilient evidence from studies explicitly implementing or evaluating noise-resilient or denoising approaches (eg, spectral subtraction, Wiener filtering, wavelet filtering denoising, and deep learning–based enhancement), and it forms the primary evidence base for assessing robustness. Tier B entails comparator evidence from studies using standard or non–noise-resilient feature extraction techniques (eg, MFCCs and spectrograms) without explicit denoising. These were included to provide baseline comparisons and to highlight the gap since many bioacoustics studies still rely on such methods despite operating under noisy conditions.

Eligible studies had to present empirical or computational results using machine learning or deep learning–based classification models and report at least one standard performance metric such as accuracy, precision, robustness, or generalizability. In addition, the review included both primary and secondary data-based studies, as long as they provided sufficient methodological details regarding feature extraction and classification pipelines.

Studies were excluded if they did not involve biological acoustic signals or if they focused solely on speech or music processing unrelated to ecological or health contexts. Review articles, theoretical discussions without implementation or evaluation, and non–peer-reviewed sources such as preprints, editorials, or technical reports were also excluded. Finally, studies that failed to describe their dataset, feature extraction process, or performance evaluation methods in sufficient detail to permit meaningful analysis were omitted.

### Protocol and Registration

The review was registered as required by PRISMA 2020 guidelines in the Open Science Framework (OSF) to enhance transparency. The review protocol was registered on August 16, 2025 (registration ID JKD5Y). The OSF record includes the prespecified objectives, eligibility criteria, data items, and the quantitative synthesis plan. Following peer-review feedback, certain objectives and research questions were refined to reduce overlap and improve clarity. These refinements did not alter the eligibility criteria, search strategy, or dataset. A deviation log has been added to the OSF record to transparently document these revisions without altering the original aims.

### Study Selection

The study selection process followed the PRISMA guidelines to ensure transparency, reproducibility, and rigor. All retrieved records from the systematic search were imported into a reference management system, where duplicates were identified and removed. The non-English studies were machine-translated using Google Translate to support screening. Both reviewers independently cross-verified the translations against the original texts to minimize misinterpretation. There was also keen attention to the selected studies to ensure that the original papers were not later published in English to avoid omissions and double entries. The studies underwent a multistage screening process. In the initial stage, two independent reviewers performed a title and abstract screening to assess initial relevance. Any differences were resolved amicably through discussion, resulting in a consensus mutually agreed upon by both reviewers, with escalation to a third reviewer if required. Studies that clearly failed to meet the inclusion criteria were excluded at this stage, and reasons were recorded.

In the subsequent stage, potentially eligible studies underwent a full-text review. Each study was assessed for methodological clarity, relevance to bioacoustics classification, use of feature extraction techniques, and evaluation in noisy or real-world conditions. Interrater reliability was assessed using Cohen κ at both screening stages. During the title and abstract screening, the reviewers achieved an observed agreement of 90.9%, corresponding to κ=0.79. At the full-text screening stage, the observed agreement was 94.7%, indicating almost perfect agreement with κ=0.89. Discrepancies at both stages were resolved through consensus. Of the 5462 records retrieved, 132 studies met the eligibility criteria and were selected for full review. The study selection process is summarized in a PRISMA flow diagram in the Results section. There are clear details on the screening process from the retrieved studies to the final selection of the sample of 132 studies for inclusion.

### Data Extraction

We used a structured data extraction process to ensure reliability and comprehensiveness in capturing relevant study characteristics. A standardized Microsoft Excel spreadsheet was developed to systematically extract key information from each included study. The extraction form was designed to align with the objectives and review questions, capturing both methodological details and performance-related data. Data extracted for each study include:

1. Bibliographic details: authors, title, and year containing basic bibliographic information to uniquely identify and reference the studies.
2. Study design and setting: Including whether the study was experimental, comparative, or simulation-based, together with the domain (clinical infant cry versus ecological) and the context (NICU, field, or lab).
3. Dataset information: whether the dataset used was primary or secondary and its size, class distribution, and description.
4. Feature extraction techniques: Specific methods such as MFCCs, spectrograms, wavelets, and LPCCs, and advanced hybrid approaches, together with their key parameters.
5. Denoising techniques: information on whether denoising was applied and which methods were used, such as spectral subtraction, Wiener filtering, wavelet denoising, and other advanced methods.
6. Models and training: the classifier models used were also identified, such as machine learning, statistical, neural networks, and deep learning models.
7. Performance metrics and contrasts: key performance metrics such as classification accuracy, precision, and *F*_1_-score, together with CIs or statistical tests if reported.
8. Application domain: the area of implementation, such as wildlife monitoring, health care, infant cry analysis, marine mammal detection, or smart sensing.
9. Deployment context: document real-world use, simulation, or proof-of-concept, and also reported challenges such as noise variability, data imbalance, or model generalizability where documented.
10. Where available, each study’s future direction or proposed improvements were also extracted to identify research gaps and emerging priorities in noise-resilient bioacoustics analysis.

The extraction was conducted independently by 2 reviewers, with cross-validation to ensure reliability. Missing or unclear information was noted, and where necessary, corresponding authors were contacted for clarification. This structured approach enabled comprehensive synthesis and comparison across studies with diverse methodologies and application contexts.

### Data Synthesis and Analysis

The extracted data were analyzed both qualitatively and quantitatively. Qualitative synthesis entailed identification of trends in noise-resilient methods, recurring challenges, emerging technologies, and synthesis of study findings to highlight advancements. On the other hand, quantitative summaries reported frequencies and distributions for classifier model classes, feature families, denoising techniques, deployment contexts, and performance metrics. Given heterogeneity in datasets, noise protocols, and outcomes, formal meta-analysis was not appropriate. We used a structured narrative approach: (1) group studies by feature, denoising family, model class, and domain (clinical vs ecological); (2) contrast performance under noise against baselines when available; (3) examine transferability and deployment evidence; and (4) integrate risk-of-bias and reporting-quality signals into interpretation.

To ensure methodological rigor and transparency, a dual quality assessment approach was adopted, combining both reporting quality and methodological bias evaluation. While the quality rating did not dictate the inclusion of studies, it aimed to present an outline of the reliability and transparency of the selected research. The TRIPOD (Transparent Reporting of a Multivariable Prediction Model for Individual Prognosis or Diagnosis) checklist was used to assess the intelligibility, completeness, and reproducibility of reporting in each study. Five key TRIPOD components were evaluated: title and abstract, introduction, methods, results, and discussion. Each component was scored as either compliant (1) or noncompliant (0), yielding a maximum possible score of 5.

The risk of bias across the studies was assessed to identify potential sources of bias in the reviewed studies. Given the machine learning focus of this review, domain-specific risk of bias criteria was applied to five core areas: (1) bias in data sources and sampling to check whether the data were representative, balanced, and appropriately selected; (2) bias in labeling and ground truth to check whether labels were accurate, consistent, and validated; (3) bias in feature extraction and preprocessing to check whether preprocessing and feature engineering introduced potential artifacts or limitations; (4) bias in model training and evaluation to check whether the training-validation-test split, metrics, and evaluation protocols were appropriately implemented; and (5) bias in reporting and interpretation of results to check whether performance was selectively reported or overly generalized. Each domain was rated as “low,” “moderate,” or “high” risk of bias. The overall risk of bias was then derived from these domain-level assessments, with a deliberate distribution.

All assessments were conducted independently by 2 reviewers with consensus resolution. Importantly, neither TRIPOD nor risk of bias ratings determined study inclusion; rather, they informed interpretation by highlighting areas of greater or lesser methodological confidence. The numerical results of TRIPOD and risk of bias assessments are reported in the Results section. By combining the TRIPOD framework appraisal with domain-specific risk of bias, the quality assessment provided a robust evaluation of the selected studies’ validity and reliability. This comprehensive approach ensured that the findings of this systematic review were built on a foundation of transparent, high-quality research.

## Results

### Study Selection

**Figure 1.**
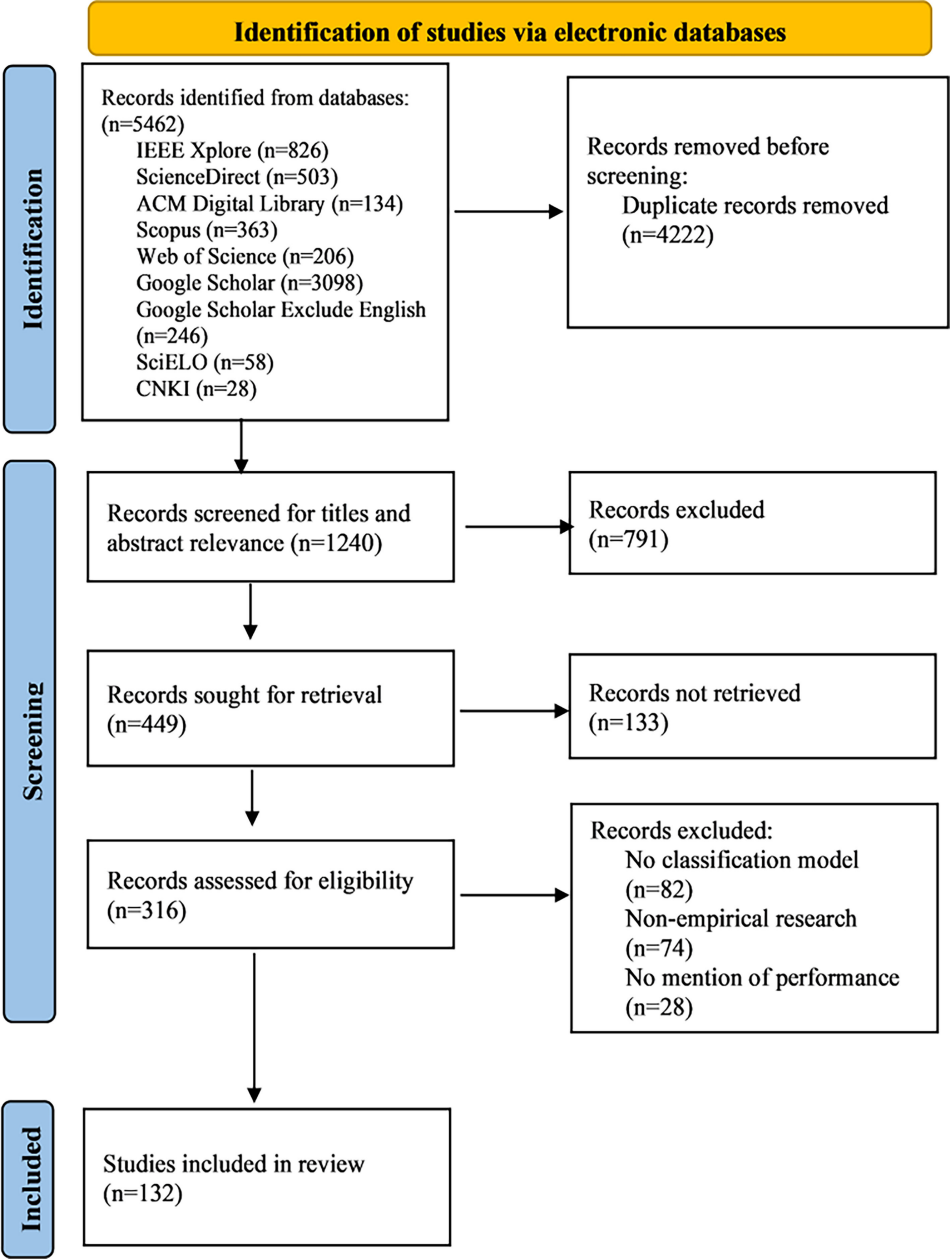
PRISMA (Preferred Reporting Items for Systematic Reviews and Meta-Analyses) flowchart.

This review synthesized 132 studies (Multimedia Appendix 3) published between 2003 and 2024, spanning two primary application domains: ecological monitoring studies (n=80, 60.6%) and clinical infant cry studies (n=52, 39.4%). The study selection process is summarized in a PRISMA flowchart in Figure 1. Studies were further stratified into tier A, comprising noise-resilient pipelines with explicit denoising or robustness testing strategies, 47% (n=62) of studies, and tier B, comprising comparator pipelines without explicit denoising, 53% (n=70) of studies. This distribution highlights both the predominance of ecological applications and the substantial proportion of studies still relying on non–noise-resilient baselines. To establish the reliability of the evidence base, we first summarize the outcomes of the reporting quality (TRIPOD) and risk of bias assessments. Findings are then presented in 5 sections: research focus, methodological trends, performance outcomes, deployment and cross-domain transferability, and limitations with future priorities.

### Reporting Quality and Risk of Bias

The TRIPOD checklist revealed that all reviewed studies (n=132) demonstrated excellent reporting standards, achieving a perfect compliance score (5/5, 100%). This clearly indicates that titles and abstracts, introductions, methods, results, and discussions were consistently reported in line with transparency standards. This reflects a strong cultural shift in the bioacoustics and audio classification community toward structured and reproducible reporting practices. While TRIPOD compliance was universal, a high score largely captures surface-level reporting standards (eg, presence of sections and completeness of description) rather than deeper methodological rigor. In practice, studies varied in how clearly they justified feature extraction choices, described preprocessing, or documented evaluation protocols. This suggests that, although reporting has become standardized, interpretive caution is still required when assessing methodological robustness.

The risk of bias evaluation across all included studies revealed strong methodological rigor overall, with most domains rated as low risk. However, a small proportion of studies exhibited moderate or high risks in specific areas. The risk of bias assessment results across the studies in each domain are presented in Table 1.

**Table 1.** Risk of bias values across various domains.

Risk of bias	Low	Moderate	High
Bias in data sources and sampling	127	3	2
Bias in labeling and ground truth	127	3	2
Bias in feature extraction and preprocessing	126	2	4
Bias in model training and evaluation	125	5	2
Bias in reporting and interpretation of results	130	0	2
Overall risk of bias	116	10	6

Bias in data sources and sampling was rated low in 96.2% (127/132) of the studies, indicating that the studies used clearly documented datasets with appropriate sampling strategies. However, 2.3% (3/132) [39-41] and 1.5% (2/132) [42,43] were rated as moderate and high variability due to a lack of clear discussion on sample size and sample selection strategies.

Bias in labeling and ground truth was rated low in 96.2% (127/132) of the studies, reflecting strong adherence to consistent annotation practices. Bias in feature extraction and preprocessing was rated low in 95.5% (126/132) of the studies, suggesting a high degree of transparency in preprocessing protocols. However, 1.5% (2/132) [44,45] and 3.4% (4/132) [12,46-48] were rated as moderate and high, largely due to a lack of justification for chosen features and unclear preprocessing steps.

Bias in model training and evaluation was rated low in 94.7% (125/132) of the studies, demonstrating widespread adoption of sound training practices. A small proportion (5/132, 3.8%) [49-53] were rated as moderate, while 1.5% (2/132) [35,54] were rated as high, due to improper validation schemes and related design weaknesses.

Bias in reporting and interpretation was rated low in 98.5% (130/132) of the studies, indicating that most studies provided transparent and well-supported results. However, 1.5% (2/132) [55,56] were rated high, mainly due to lack of clarity in reporting key results.

Overall risk of bias was rated low in 87.9% (116/132) of the studies, highlighting the generally high methodological rigor across the reviewed literature. A notable proportion of 7.6% (10/132) [40-44,50-52,57,58] were rated as moderate, while 4.5% (6/132) [12,35,48,54-56] were rated as high, often due to cumulative concerns across multiple bias domains. Detailed per-study risk of bias ratings can be found in Multimedia Appendix 4. Although the corpus is predominantly low risk of bias, the small cluster of moderate or high ratings concentrates in preprocessing justification and evaluation rigor. In subsequent results, we interpret performance and robustness claims with greater weight placed on low-risk studies and flag results from studies with methodological gaps where relevant.

### Trends and Research Focus

**Figure 2.**
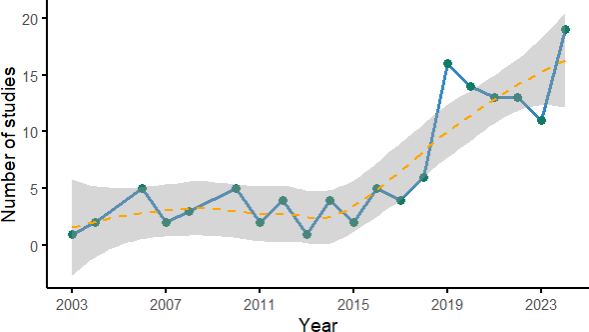
Trend of the number of publications per year.

Research in noise-resilient bioacoustics has expanded rapidly since 2019, with most contributions centered on model innovation, while noise robustness and deployment remain underrepresented. The reviewed studies reflect a growing momentum in the field of noise-resilient bioacoustics, demonstrated by a pronounced upward trend in publications over the past decade. The annual trend distribution in Figure 2 illustrates steady growth, with a notable increase in the number of publications from 2019 onward, peaking in 2024 with 14.4% (19/132). This surge coincides with the uptake of deep learning and larger annotated datasets.

Overall, 65.2% (86/132) were published between 2019 and 2024, highlighting a recent surge in research activity motivated by advancements in machine learning, particularly deep learning architectures, and an increased availability of publicly accessible, annotated acoustic datasets. This rapid expansion underscores the field’s responsiveness to technological innovation and its potential for addressing practical challenges. The field is shifting adeptly from merely experimental exploration to a mainstream research agenda in ecological monitoring, wildlife conservation, and infant cry monitoring.

In terms of contribution types, the vast majority of studies (112/132, 84.8%) focused on model innovation, primarily through the design of novel architectures and algorithms for bioacoustics classification. These included deep learning approaches such as CNNs, RNNs, CRNNs, and transformer-based models. Hybrid frameworks combined traditional signal processing techniques, for example, MFCCs and spectral features, with neural networks. Within this category, some studies emphasized architectural novelty, for example, attention mechanisms and temporal-context modeling, while others explored optimization strategies such as regularization, hyperparameter tuning, or multimodal feature fusion. Feature selection and engineering were addressed in 43.9% (58/132) of studies, emphasizing the role of extracting relevant and informative features to improve classification accuracy.

Noise robustness and generalization were explicitly explored in 28.8% (38/132) of studies, which incorporated denoising techniques, noise-aware training, and evaluation across diverse acoustic environments to improve real-world performance. Finally, only 14.4% (19/132) of studies reported deployment-focused applications, demonstrating implementations in wildlife conservation zones, smart farming, NICUs, and edge-based monitoring systems.

The field remains heavily weighted toward architectural innovation, with robustness testing and deployment underrepresented. This imbalance highlights a translational gap—methodological advances are plentiful—but their practical application in real-world bioacoustics is still emerging.

### Methodological Landscape

The methodological landscape across the reviewed studies showcases a strong emphasis on empirical evaluation, consistent with the practical and performance-driven nature of noise-resilient bioacoustics research. Every study was categorized as experimental, involving the development, training, and testing of machine learning and signal processing models on bioacoustics datasets. The models were carefully developed, and their performance was evaluated to assess model generalization. In addition to an experimental foundation, 36.4% (48/132) were comparative studies, systematically benchmarking multiple models or feature extraction pipelines under controlled noise conditions. These studies were instrumental in benchmarking traditional versus advanced techniques and identifying optimal configurations for noisy environments.

A small subset (20/132, 15.2%) of studies were also descriptive, providing detailed explanations of the models they implemented alongside empirical evaluations. This is vital for the growing research and learning era. New researchers are able to learn from what has already been done to implement improvements. Across all methodological types, studies demonstrated a commitment to reproducibility, with datasets and detailed parameter settings provided. However, the lack of standardized evaluation frameworks and consistent reporting practices remains a limitation, hindering comparability across studies. It is therefore evident that empirical research has matured broadly, but there is a continuing need for standardized methodologies to enhance comparability and real-world applicability.

### Feature Extraction Techniques

Feature extraction was nearly universal across 96.2% (127/132) of studies, with cepstral features forming the foundation of most bioacoustic classification pipelines. Spectral, temporal, and wavelet-based features served complementary roles. The distribution of the feature extraction methods across the studies per domain is presented in Table 2. The percentage distribution of each feature category in relation to the domain, as well as the tier category, is also presented to show relative variation between the domains.

**Table 2.** Distribution of feature extraction methods across the studies per domain (N=132).

	Number of studies, n (%)				
Feature type	Tier A	Tier B	Infant cry	Ecology	Total
Cepstral features	36 (27.3)	37 (28)	39 (29.5)	34 (25.8)	73 (55.3)
Filter bank and spectral representations	36 (27.3)	29 (22)	8 (6.1)	57 (43.2)	34 (25.8)
Spectral features	26 (19.7)	15 (11.4)	11 (8.3)	30 (22.7)	41 (31.1)
Temporal or time domain features	17 (12.9)	21 (15.9)	21 (15.9)	17 (12.9)	38 (28.8)
Prosodic features	8 (6.1)	8 (6.1)	5 (3.8)	11 (8.3)	16 (12.1)
Wavelet features	9 (6.8)	1 (0.8)	4 (3)	6 (4.5)	10 (7.6)

Cepstral features were the predominant category used (73/132, 55.3%), with MFCCs alone appearing in 50.8% (67/132) of studies. These features were widely favored for their ability to capture perceptually relevant sound components, closely aligned with human auditory perception. Variants such as LPCCs, constant-Q cepstral coefficients, and gammatone cepstral coefficients, often enhanced with derivatives (Δ, ΔΔ) and feature fusion strategies, were also used. Of the 132 studies, infant cry consisted of 39 (29.5%) studies, while ecology consisted of 34 (25.8%) studies. The distribution was nearly equal, indicating that the use of cepstral features in both domains was broadly comparable across applications and tiers.

Filter bank and spectral representations were also common, being used in 34 (25.8%) of the 132 studies. However, the use of these features was skewed toward the ecological domain, showing that ecological studies used these presentations in their modeling. Spectral features (38/132, 28.8%), including spectral centroid, roll-off, and entropy, quantified frequency energy distributions and were valuable for detecting anomalies in vocalizations. Similarly, the use of spectral features was skewed toward ecological application. Temporal features (41/132, 31.2%), such as zero-crossing rate, root mean square energy, and voicedness, captured time-domain behaviors and proved particularly useful in infant cry analysis for identifying cry phases and sharp transitions. Prosodic features (16/132, 12.1%) focused on pitch and intonation contours, offering insights into emotional or health-related states. Wavelet-based features (10/132, 7.6%), derived from transformations such as the discrete wavelet transform or the wavelet packet transform, were used to capture transient and nonstationary characteristics, particularly enhancing noise robustness in ecological monitoring tasks.

A small subset (n=5) [27,56,59-61] of the studies did not explicitly report a predefined feature extraction step but instead relied on the model architecture itself to learn and extract relevant features directly from the raw waveform. These approaches typically use end-to-end deep learning models, such as raw waveform CNNs, which are designed to learn spectral and temporal representations directly from the audio signal during training. These techniques are essential when developing a fully automated pipeline. However, end-to-end waveform learning without explicit feature extraction raises concerns regarding interpretability, computational cost, and data requirements.

When comparing domains, infant cry studies leaned heavily on cepstral and prosodic features, reflecting the speech-like and emotionally driven nature of cries. MFCCs and intonation contours were most frequently used to capture subtle variations in vocal tone linked to health or emotional states. In contrast, ecological monitoring studies applied a broader mix of spectral, temporal, and wavelet features to represent the diversity of animal calls and environmental soundscapes. These choices highlight the domain-driven adaptation of feature extraction. It was evident that studies in tier A concentrate on filter bank, log mel, and spectral descriptors. These feature families align perfectly with denoising applications. Tier B studies, however, inflate cepstral feature use since there was no explicit denoising.

Feature extraction emerged as a cornerstone of noise-resilient bioacoustics classification. Cepstral features dominate current practice, while spectral, temporal, and prosodic features provide complementary insights. Wavelets offer noise-robust representations, and end-to-end models mark an emerging direction toward automation. Together, these approaches illustrate a balance between established feature engineering and exploratory deep learning–based representation learning.

### Denoising Techniques

**Figure 3.**
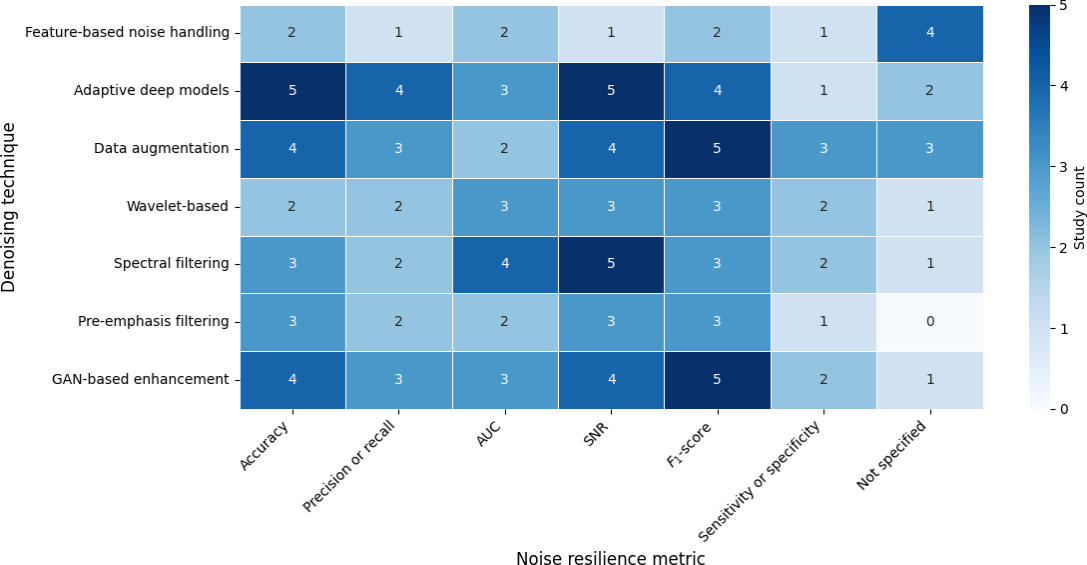
Distribution of noise-resilient metrics and denoising application across the studies. AUC: area under the receiver operating characteristic curve; GAN: generative adversarial network; SNR: signal-to-noise ratio.

Nearly half (62/132, 47%) of the reviewed studies presented denoising application in the modeling pipeline tier A, and more than half (76/132, 57.6%) of the studies presented use of a noise-resilient metric to assess model robustness. While traditional signal processing methods remain common, advanced deep learning–based denoising is gaining traction, though still underrepresented. A visual representation of the distribution of these methods is presented in Figure 3.

Darker shading indicates higher study counts. Adaptive deep models and GAN-based enhancement were most frequently paired with robust evaluation metrics such as *F*_1_-score and SNR degradation, while classical approaches (eg, pre-emphasis and spectral filtering) relied more on accuracy and AUC alone. Studies omitting denoising often reported only accuracy, highlighting a reporting gap between baseline pipelines and noise-resilient methods.

Traditional denoising approaches rooted in classical signal processing were used in 25% (33/132) of studies. These techniques included pre-emphasis filters to suppress low-frequency noise, spectral subtraction, Butterworth high-pass filters, and windowing techniques. Adobe Audition and WavePad Sound Editor were also used for manual noise reduction and audio cleanup. Transformations such as the fast Fourier transform, discrete wavelet transform, and wavelet packet transform were leveraged to enhance feature robustness against noise, together with energy-based descriptors like root mean square energy, zero-crossing rate, and segmentation techniques that also supported noise minimization.

Advanced deep learning–based denoising techniques were used in 16.7% (22/132) of the studies, marking a shift toward more adaptive and context-aware noise handling. These approaches included the use of stage-wise GANs for structured denoising [62], PCEN for real-time noise suppression [3], and deep CNNs trained with a pretext to enhance resilience [63]. A portion of the studies used contextual metadata-aware CNNs [56], dimensionality reduction via YAMME [50], or custom neural denoisers like DS-Denoiser [63] and Burn Layer noise injection strategies [48].

In 12.9% (17/132) of studies, noise resilience was achieved indirectly through strategic feature design and training methodologies rather than explicit denoising. These included data augmentation with controlled noise injection [64], spectrogram normalization [65], entropy-based descriptors, and frame-based segmentation to reduce the impact of transient background noise [66]. Several studies introduced false-positive distractors during training to improve model discrimination [35,55], while others used SNR-aware evaluation metrics [35,67] and principal component analysis to filter out irrelevant variation [68].

Noise-resilient metrics included standard evaluation tools such as AUC, *F*_1_-score, precision, recall, and accuracy, often reported across multiple SNR levels (eg, 100 dB, 3 dB, 0 dB, and −3 dB) to capture degradation effects [35,69,70]. Some studies used equal error rate or Earth Mover’s Distance to assess alignment between predictions and ground truth under distortion [65]. Studies also introduced custom fitness metrics that weighted false positives caused by noise more heavily or used domain-specific indicators like perceptual evaluation of speech quality and false alarm rates [56]. These metrics were crucial for evaluating not just raw classification accuracy, but also how robustly the models maintained performance in realistic and adverse audio conditions.

Denoising strategies also diverged across domains. Infant cry studies often applied classical noise-reduction methods such as spectral subtraction and Wiener filtering to handle consistent background noise in hospitals or home environments. More recent works explored denoising autoencoders to improve robustness in clinical deployment. Ecological monitoring, by contrast, dealt with far more heterogeneous noise sources, including overlapping species, wind, and rain. As a result, adaptive filtering and multiband denoising approaches were common, enabling resilience to highly variable outdoor acoustic conditions. The ecological field has gone into extreme detail to ensure features for model development are not affected by environmental noise.

It is evident that the clinical field is heavily dependent on classical denoising and occasionally AUC and *F*_1_-score metrics, while robustness testing was less frequent. However, in ecology, there is greater use of deep or indirect denoising and systematic evaluation across SNR levels, reflecting highly variable outdoor noise. Noise-resilient evaluation was largely confined to tier A pipelines. Studies that skipped denoising (tier B) also rarely reported robustness metrics, inflating apparent performance. Infant cry pipelines showed limited robustness testing, while ecology studies drove innovation in both denoising and noise-resilient evaluation frameworks.

### Classifier Architectures and Performance

A diverse range of classification models was used across the reviewed studies, reflecting both the evolution of machine learning techniques and the complexity of bioacoustics data. The distribution of classifier architectures is presented in Table 3.

**Table 3.** Distribution of classifier architectures per domain (N=132).

	Number of studies, n (%)				
Model family	Tier A	Tier B	Infant cry	Ecology	Total
Traditional machine learning	27 (20.5)	20 (15.1)	23 (17.4)	24 (18.2)	47 (35.6)
CNN^a^	18 (13.6)	21 (15.9)	7 (5.3)	32 (24.2)	39 (29.5)
CRNN^b^ or hybrid	2 (1.5)	2 (1.5)	2 (1.5)	2 (1.5)	4 (3)
Deep neural network	13 (9.8)	13 (9.8)	7 (5.3)	19 (14.4)	26 (19.7)
Classical neural network	2 (1.5)	10 (7.6)	11 (8.3)	1 (0.8)	12 (9.1)
Transformer	0 (0)	1 (0.8)	0 (0)	1 (0.8)	1 (0.8)

a CNN: convolutional neural network.

b CRNN: convolutional recurrent neural network.

Traditional machine learning architectures dominated the reviewed literature in both ecological monitoring and infant cry analysis. More than half of the studies (70/132, 53%) reported accuracies ≥90%, with CNN-based approaches most frequently associated with high performance. Traditional models such as support vector machines (SVMs), *k*-nearest neighbors, decision trees, Gaussian mixture models, and Naive Bayes were used in 48.5% (64/132) of the studies, with SVMs being used in 24.2% (32/132) of the reviewed studies. These models typically relied on handcrafted features like MFCCs and LPCCs and showed decent performance under low-noise or controlled conditions but often struggled in the presence of complex noise or overlapping signals.

In contrast, deep learning models appeared in 53% (70/132) of the studies and formed the dominant category. CNNs, RNNs, long short-term memory, and their hybrids (eg, CRNNs) were frequently used since they can automatically learn features from raw data. Advanced models—ResNet, EfficientNet, and DenseNet—offered high performance with transfer learning advantages. The CNN model was used in 42.4% (56/132) of the studies.

Classical neural networks, including multilayer perceptrons, time-delay neural networks, and probabilistic neural networks, were seen in 22.7% (30/132) of studies, while 24.2% (32/132) used hybrid or ensemble models, such as CNN + RNN architectures or transformer-based pipelines. These advanced approaches were particularly suited for handling real-world noise, variability in signal patterns, and generalizing across datasets, making them ideal for deployment in bioacoustics monitoring systems.

The performance of these models was centered on classification accuracy, with several studies also reporting precision, recall, and *F*_1_-score. Most studies (96/132, 72.7%) reported classification accuracies exceeding 85%, with 53% (70/132) achieving 90% or higher. High-accuracy models were typically based on deep learning architectures, particularly CNNs, CRNNs, and transformer variants. Accuracy was generally enhanced when models incorporated noise-aware training, denoising preprocessing, or attention mechanisms.

Models using traditional machine learning techniques (eg, SVMs and decision trees) tended to report lower accuracies, often in the 70%‐85% range, especially when tested under real-world acoustic conditions. However, in low-noise or synthetic scenarios, these models performed comparably well. In studies that evaluated precision and recall, scores were typically balanced, often above 0.8, especially in binary classification tasks. However, multiclass classification scenarios showed slightly reduced precision in species-rich datasets, often due to class imbalance or overlapping vocalizations.

Studies leveraging ensemble methods or hybrid networks showed some of the best overall performance, with AUC values as high as 0.96 and accuracy consistently above 92% when evaluated on diverse and noisy bioacoustics datasets. Notably, some studies used post hoc statistical analysis such as the Nemenyi test, ANOVA, or CIs to validate model significance across different noise conditions or experimental configurations.

**Figure 4.**
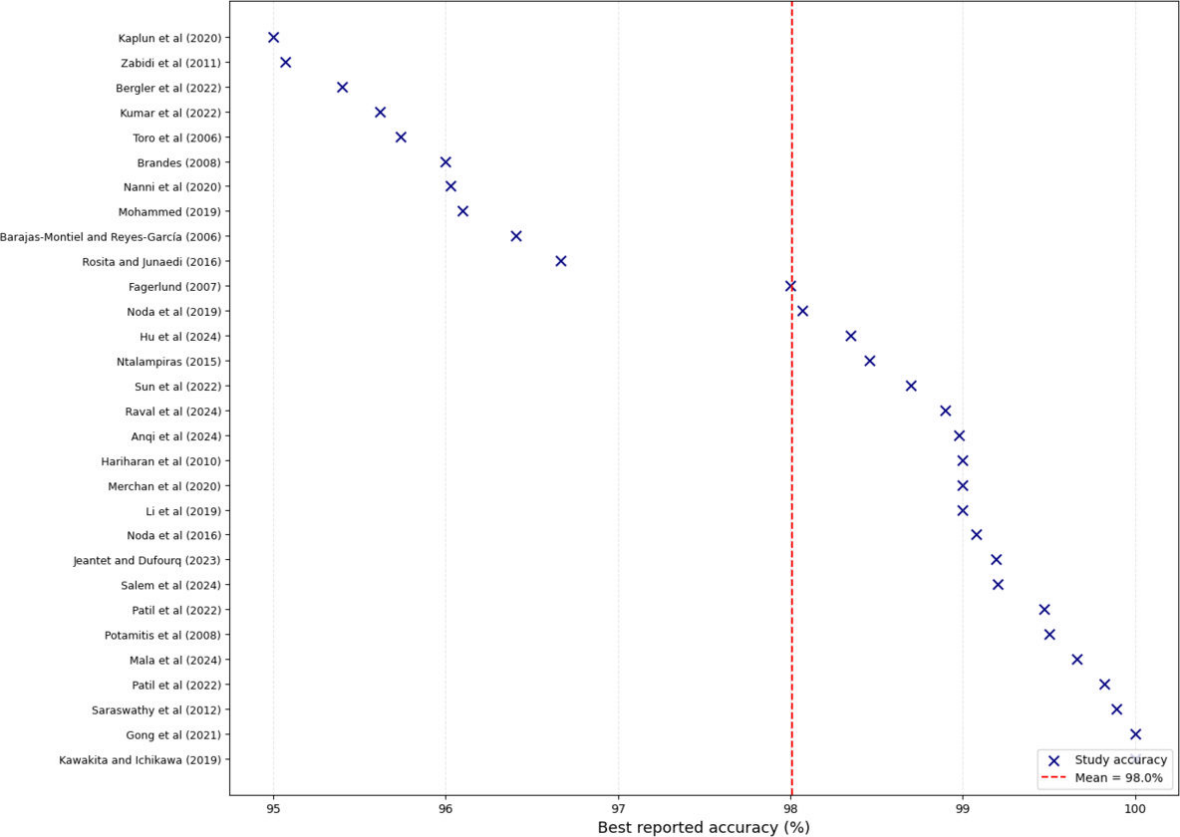
Forest plot of best-reported accuracies reporting the top 30 studies [27,51,58,64,66-91].

A forest plot of the best-reported accuracies in Figure 4 illustrates the performance clustering of the majority between 95% and 100%. This reflects the strong classification potential of modern bioacoustics models across domains. The clustering near 99% indicates a ceiling effect in reported results. Most of these values originate from tier B pipelines evaluated under clean or synthetic conditions, while tier A pipelines tested under noisy ecological conditions reported more variable results (approximately 75%‐95%). This discrepancy highlights that reported best-case accuracies often reflect optimized conditions rather than real-world robustness.

Infant cry studies often reported >95% accuracies under controlled conditions, while wildlife monitoring required more extensive preprocessing or noise-handling strategies to achieve comparable results. These findings highlight both the promise of bioacoustics classification and the need for standardized reporting of performance variability across noise levels and datasets.

**Figure 5.**
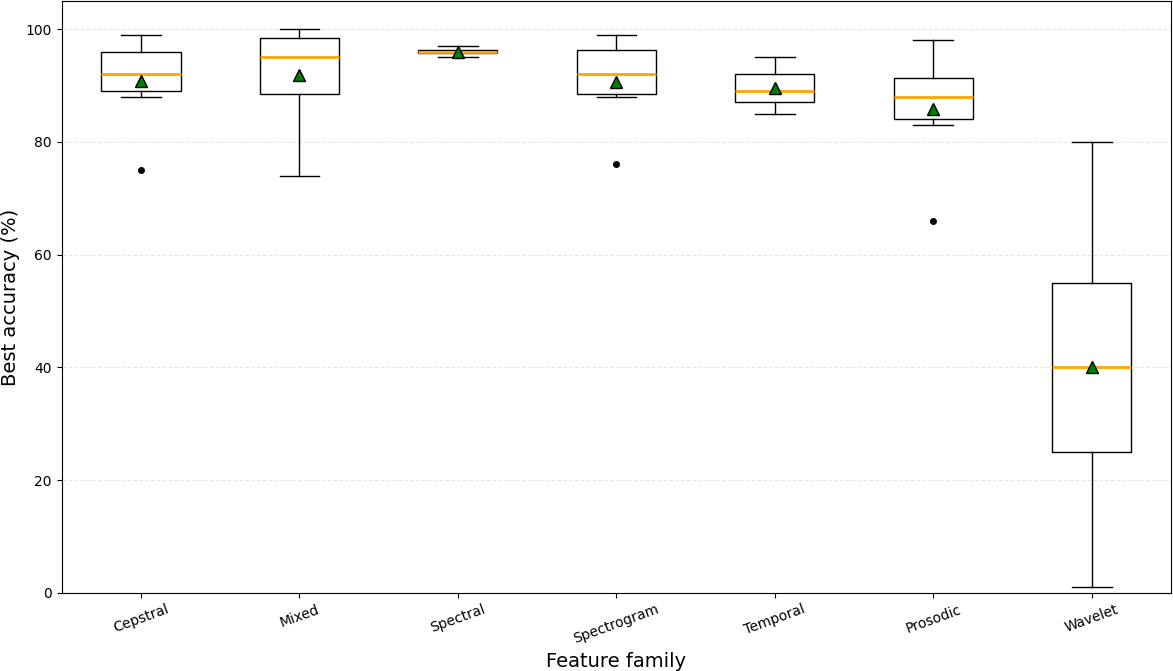
Distribution of accuracy against feature family.

Figure 5 compares the best-reported accuracies across feature families. Cepstral, spectrogram-based, and mixed feature sets clustered above 90%, confirming their central role in bioacoustics classification. However, tier A pipelines achieved these results under noisy conditions when using spectrogram or log-mel representations, while tier B pipelines often reported inflated accuracies from cepstral-only inputs under clean settings. Temporal features produced moderately strong outcomes but showed greater variance, particularly in infant cry studies. Wavelet-based features exhibited the greatest spread (0%‐80%), reflecting their experimental use in ecological tier A pipelines for transient, nonstationary noise. These results suggest that while cepstral and spectrogram-based features remain the most reliable overall, robustness under realistic noise depends on whether the pipeline incorporates explicit tier A resilience strategies.

In tier A pipelines, deep learning models dominated, particularly CNNs and CRNNs, which together accounted for nearly two-thirds of ecological studies. These were typically paired with noise-resilient features such as log-mel spectrograms or PCEN, enhancing robustness across variable acoustic conditions. By contrast, tier B pipelines were skewed toward traditional machine learning, and classical neural networks were most often applied with MFCCs. These models frequently reported strong results in clean or synthetic conditions, but robustness to real-world noise was rarely evaluated.

In domain comparison, infant cry studies leaned heavily on interpretable and computationally efficient approaches, with traditional machine learning used in 44.2% (23/52) of pipelines and classical neural networks in 21.2% (11/52), while CNNs were fewer, at 13.5% (7/52). Most of these pipelines were tier B baselines, reflecting a focus on clinical interpretability and resource efficiency over robustness. Ecological studies, in contrast, showed stronger adoption of CNNs, used in 40% (32/80) of pipelines, and deep neural networks, used in 23.8% (19/80), particularly within tier A pipelines. Transformers were rare and appeared only in ecology and tier B pipelines in 1.25% (1/80), reflecting early experimentation with sequence models. It is therefore evident that robust tier A ecological pipelines favored deep CNN and CRNN models with noise-resilient features, while infant cry pipelines remained anchored in tier B baselines combining MFCCs with traditional machine learning or classical neural networks. This contrast highlights a trade-off between robustness and interpretability across domains.

Performance reporting was dominated by classification accuracy, though many studies supplemented it with *F*_1_-score, precision, recall, or AUC. Most studies (96/132, 72.7%) reported accuracies ≥85%, with more than half (70/132, 53%) ≥90%. High-performing models were typically deep learning architectures (CNNs, CRNNs, and transformers). Tier A pipelines consistently tested performance under noisy conditions and reported smaller accuracy drops across SNR levels (typically 5%‐10%). Tier B pipelines rarely incorporated noise protocols and often reported inflated best-case accuracies (>95%), reflecting performance under clean or synthetic conditions rather than realistic robustness.

Infant cry studies frequently reported very high accuracies (>95%), but these were predominantly from tier B baselines using MFCC + traditional machine learning or CNN in controlled NICU or home environments. Few infant cry studies tested performance in truly noisy or cross-population conditions, limiting confidence in their generalizability. Ecological studies, by contrast, showed a wider performance spread (approximately 75%‐95%), reflecting more diverse datasets, taxa, and recording environments. Tier A ecological pipelines that incorporated denoising and spectrogram or PCEN features frequently exceeded 90% accuracy, but results were more variable due to dataset complexity and nonstationary noise. It is evident that reported accuracies cluster near ceiling values, but these reflect tier B clean-condition pipelines more than tier A robustness evidence. Infant cry studies appear stronger on paper but are less often validated under noise, whereas ecological tier A pipelines, though more variable, provide the most convincing demonstrations of resilience under realistic acoustic conditions.

### Quantitative Analysis of Performance

Of the 132 included studies, 82.6% (n=109) reported classification accuracy, while 21.2% (n=28) reported *F*_1_-scores. Accuracy was the dominant performance indicator, particularly in traditional and early deep learning approaches, whereas *F*_1_-score appeared more often in recent studies emphasizing class balance in imbalanced datasets. Across all studies, the mean accuracy was 89.47% (SD 30.82%) with a median of 93.64%, ranging from 2% to 343%, while the mean *F*_1_-score was 93.04% (SD 7.86%) with a median of 95.88%, spanning 71%‐100%. These results indicate generally high predictive capability across bioacoustic classification models, though the wide variation in accuracy reflects methodological diversity in dataset size, preprocessing techniques, and evaluation strategies. The overall distribution of quantitative findings is presented in Table 4.

**Table 4.** Summary of performance metrics across tier A and tier B studies.

Metric	Tier	Studies, n	Mean, %	Median, %	SD	Min	Max	95% CI (lower-upper)	*P* value (versus tier A)
Accuracy	A	52	83.64	90.5	22.51	2	100	77.6‐89.7	Reference
Accuracy	B	57	94.79	94	36.19	20	343	85.3‐104.3	.04
*F*_1_-score	A	9	93.49	92.6	5.35	87	100	89.1‐97.8	Reference
*F*_1_-score	B	19	92.83	98	8.93	71	100	88.6‐97.1	.67

Statistical comparison revealed a significant difference in accuracy between the 2 tiers (*P*=.04), confirming that tier B models achieve superior accuracy overall. However, no significant difference was observed in *F*_1_-scores (*P*=.67), suggesting that while denoising enhances general classification accuracy, it does not consistently alter the precision-recall trade-off.

**Figure 6.**
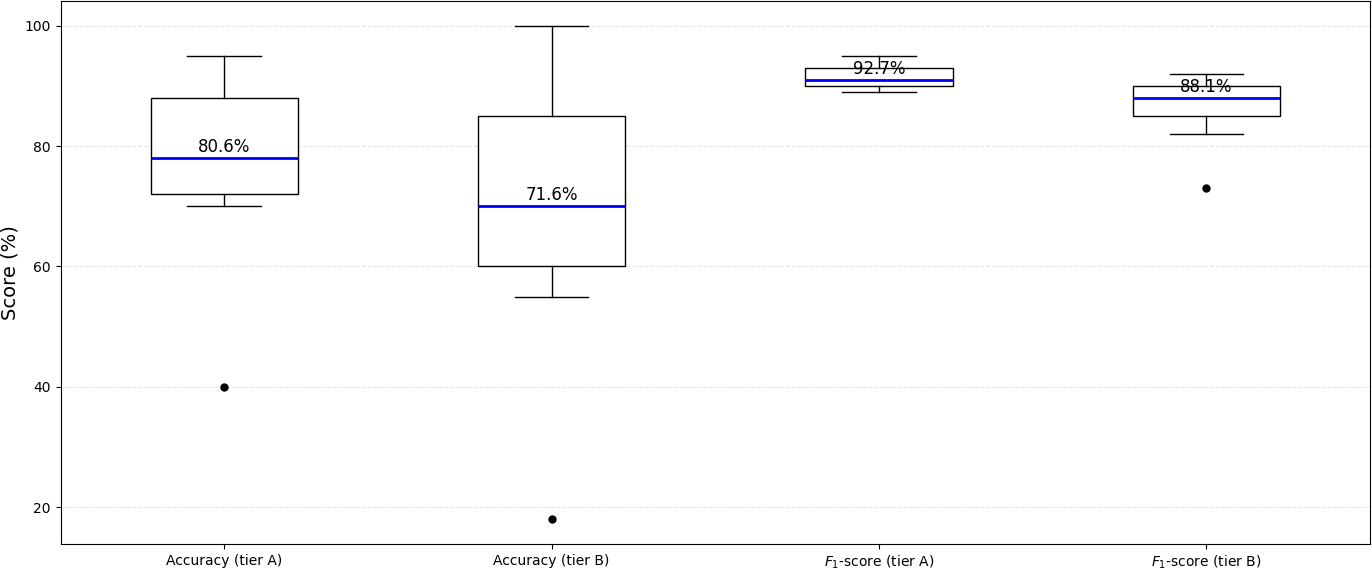
Distribution of model accuracy and *F*_1_-score across tier A and tier B studies.

Tier A models achieved a higher mean accuracy (94.79%) compared to tier B (83.64%), suggesting the benefit of integrating denoising and noise-resilient feature extraction methods. However, tier B exhibited greater variability (SD 36.19) than tier A (SD 22.51), indicating that while noise-resilient models often achieve superior results, their performance may depend heavily on implementation quality and dataset characteristics. The *F*_1_-scores of both tiers were relatively consistent, averaging 93.49% for tier A and 92.83% for tier B, implying that noise handling primarily improves robustness rather than precision-recall balance. The comparative distribution of performance metrics across tiers A and B is illustrated in Figure 6. The box plot summarizes the spread and central tendency of both accuracy and *F*_1_-score values, highlighting that denoising generally elevates overall performance yet increases score variability. The clear clustering of *F*_1_-scores around the upper quartile further confirms the stability of the precision-recall balance across studies.

In domain-specific comparison, infant cry research reported a higher overall mean accuracy of 92.8% and a mean *F*_1_-score of 94.6%, with relatively low variability, reflecting the controlled recording settings, smaller class counts, and limited background interference characteristic of clinical datasets. In contrast, ecological studies exhibited broader score dispersion, with accuracy values ranging from 70% to 95% and *F*_1_-scores between 80% and 96%, indicating greater heterogeneity due to environmental noise, overlapping species vocalizations, and larger taxonomic class sets. Tier B (noise-resilient) ecological studies achieved modest gains in mean accuracy (+4.2%) compared to tier A, though with higher standard deviation, underscoring the impact of denoising complexity in natural soundscapes. Conversely, infant cry models benefited less from denoising interventions, maintaining stable performance even under tier A configurations.

### Deployment and Application Domains

**Figure 7.**
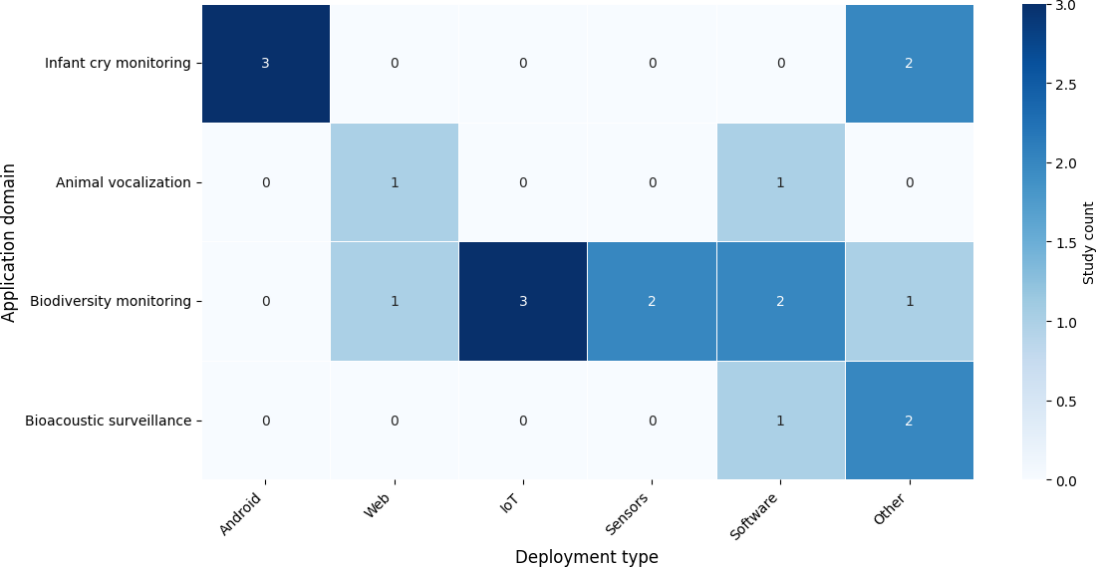
Distribution of deployment across various bioacoustic domains. IoT: Internet of Things.

A small number of studies (19/132, 14.3%) discussed the potential deployment of models in real-world environments. Figure 7 shows how these studies were deployed in various bioacoustic domains. It shows that infant cry deployments were primarily smartphone-based caregiver tools, whereas ecological applications emphasized the Internet of Things (IoT) and sensor networks for biodiversity monitoring. Bioacoustic surveillance deployments were rare. Importantly, almost all deployment cases arose from tier A studies, underscoring that robustness evidence is a prerequisite for translation.

A significant portion (7/132, 5.3%) involved tool-based deployments, including Android apps [92,93], web interfaces [64,88], and user-friendly platforms for real-time monitoring and human verification [13,71,94,95]. IoT and embedded systems were featured in 4.5% (6/132) of studies, leveraging low-power devices [27,49,96,97], sensor networks [3,72], and mobile hardware for field applications. Open-source software [98] solutions such as DeepSqueak [63] and ORCA-SPOT [99] were used in 2.3% (3/132) of studies, and 1.5% (2/132) of studies used limited offline toolkits such as MATLAB’s Neural Network Toolbox [100,101]. The deployed studies were distributed across the various applications of bioacoustics classification technologies. Infant monitoring systems (5/132, 3.8%) focused on detecting cries associated with health conditions or needs, supporting early diagnosis and caregiver response.

Animal vocalization monitoring (2/132, 1.5%) aimed to detect and classify specific species calls, contributing to behavioral and ecological research. Biodiversity monitoring (9/132, 6.8%) represented the largest category, with deployments targeting broad-scale species tracking, conservation efforts, and habitat assessment in diverse ecosystems. Lastly, bioacoustics surveillance (3/132, 1.7%) focused on monitoring environmental soundscapes for human-induced or unusual acoustic events, supporting real-time situational awareness and management in protected or sensitive areas.

Infant cry deployments (5/132, 3.8%) focused on caregiver and clinical support, such as smartphone apps and hospital monitoring tools. These pipelines emphasized real-time cry detection for diagnosis and caregiver response but were limited by data privacy, ethical constraints, and the need for interpretability. Ecological deployments (14/132, 10.6%) concentrated on scalability, leveraging IoT and embedded systems for biodiversity monitoring, conservation surveillance, and edge-based species detection. Tools such as DeepSqueak and ORCA-SPOT exemplified open-source tier A systems tailored to diverse and noisy outdoor environments.

Deployment patterns further underline the distinct priorities of each domain. Infant cry research emphasized caregiver support through hospital tools and smartphone apps, focusing on real-time cry detection and monitoring for clinical or home use. Ecological monitoring prioritized scalability, leveraging IoT sensor networks, embedded low-power devices, and open-source tools such as ORCA-SPOT for biodiversity tracking. Whereas infant cry deployments aim for individualized, human-centered decision support, ecological deployments are oriented toward large-scale, automated monitoring across ecosystems. It was also evident that deployed studies are a portion of tier A, underscoring that noise robustness is a precondition for real-world deployment. Deployment in the real world is directly related to denoising, and studies with no implicit denoising did not translate to deployment.

Despite these promising efforts, a primary limitation reported across studies was the lack of large, high-quality, standardized datasets for both clinical and ecological domains. This gap restricted generalization, with most deployments validated in narrow or pilot settings. Failures and constraints were often tied to dataset variability, hardware limitations, and energy efficiency trade-offs, underscoring the need for more robust field trials, benchmark datasets, and harmonized evaluation protocols to achieve sustainable real-world applicability.

Beyond technical feasibility, deployment in sensitive domains requires attention to ethical, interpretability, and infrastructural concerns. In neonatal care, noise-resilient models must safeguard patient privacy and provide transparent outputs that clinicians and caregivers can trust. Similarly, ecological monitoring systems need explainable decisions to ensure transparency in conservation policy and sustainability of automated surveillance. These considerations highlight that deployment success depends not only on accuracy but also on responsible integration into clinical and environmental workflows.

### Challenges and Future Direction

Despite significant advancements in noise-resilient bioacoustics classification, several recurring challenges continue to hinder progress. A primary limitation reported across studies was the lack of large, high-quality, and standardized datasets, mentioned in 34.8% (46/132) of studies. Researchers relied on small datasets, which limited the generalizability of findings and the ability to compare models across studies. Diverse audio samples were unavailable for various species in varying recording environments, therefore restricting models from performing reliably in real-world scenarios. In addition to the limited data available, datasets were small and imbalanced, which contributed greatly to biased and overfit models.

Noise interference and acoustic variability were mentioned as a challenge in 20.5% (27/132) of studies. Studies highlighted the difficulty of extracting clean signals in field conditions, especially with background noise from human activity, equipment, or other animals. Despite some attempts using denoising and noise-aware training, many models struggled to maintain robustness under nonstationary and low signal-to-noise conditions. Additionally, inconsistencies in labeling arising from semisupervised annotations introduced noise into ground truth data, reducing model reliability.

Several deep learning approaches, especially CNNs and hybrid models, required high-performance computing resources, posing a challenge for real-time deployment. High computational costs and dependence on platform-specific tools also posed barriers to scalable and accessible deployment. Deployment was observed in very few studies despite the advancements in technology. This is due to hindrances by a lack of platform compatibility, difficulty integrating models into systems, and challenges related to real-time processing, energy efficiency, concerns over hardware requirements, and user-friendliness.

A portion of the studies (11/132, 8.3%) reported cases of overfitting, especially due to limited data for complex models, while 6.1% (8/132) reported inconsistencies in data due to variability in signal quality by recording instruments. Other challenges reported were domain transfer challenges with models trained on one species, lack of open set recognition, and false positives in some models.

Infant cry studies were constrained by small, private datasets due to ethical and privacy concerns, limiting cross-population generalizability. Ecological monitoring faced challenges with data imbalance, as rare species were underrepresented, and annotation required expert input. Both domains therefore underscore the urgent need for larger, standardized, and openly available datasets, but with differing solutions: ethical data-sharing frameworks for infant cries versus coordinated biodiversity databases for ecological monitoring.

Looking forward, many studies have emphasized the need to expand datasets across taxa, habitats, and call types, especially for underrepresented classes such as infant cries from pathological conditions to rare animal vocalizations [13,102,103]. Researchers also recommend developing semisupervised and unsupervised labeling strategies to reduce annotation burden, improving noise robustness through signal enhancement modules [73,104]. In addition, transfer learning, domain adaptation, and transformer-based architectures were proposed for better generalization [74,75]. Several studies proposed real-time deployment strategies, calling for lightweight, energy-efficient models suitable for edge computing environments [55,63].

Finally, researchers highlighted the importance of open-set recognition, anomaly detection in dynamic acoustic environments, and model interpretability, especially in health care or conservation settings. Incorporating animal-independent denoising mechanisms, optimizing data augmentation for species-specific acoustics, and refining clustering techniques for individual or dialect-level recognition were among the key future directions. Together, these efforts aim to make bioacoustics systems more scalable, reliable, and ecologically meaningful, ultimately enabling widespread deployment in biodiversity monitoring, pest detection, and early diagnosis of health conditions.

## Discussion

### Principal Findings

This systematic review synthesizes evidence from 132 studies on noise-resilient bioacoustics classification and provides an integrated perspective across methodologies, performance outcomes, and deployment contexts. The central finding is that high reported accuracies do not necessarily equate to robustness. Instead, robustness emerges from tier A pipelines—those combining explicit denoising or resilience testing with modern feature representations and architectures. By contrast, tier B pipelines, though numerous, often reported near-perfect accuracies under clean conditions but rarely progressed toward real-world deployment. This distinction frames our interpretation of the evidence against the review objectives.

### Methodological Advances

Recent years have seen a shift from handcrafted features and statistical classifiers toward deep architectures capable of capturing temporal and spectral dependencies. CRNNs, CNNs, and in some cases, transformers consistently outperformed classical machine learning under noise [76,105], echoing trends in both ecoacoustics [17] and audio enhancement research [10]. However, our synthesis shows that the real methodological gap lies not in model availability but in evaluation design. Tier B pipelines often prioritized architectural novelty but omitted robustness testing, inflating performance claims. Tier A studies, while fewer, demonstrated that rigorous evaluation across SNR levels or noise-injected datasets yields more credible, if variable, results [106]. This confirms that methodological progress in bioacoustics must be judged not only by model choice but also by the framework of validation.

### Feature Extraction and Denoising

Feature use reflected domain priorities: infant cry pipelines emphasized cepstral and prosodic features for speech-like cues [107], while ecological pipelines favored spectrogram, filter bank, and wavelet features to capture diverse soundscapes [77]. Importantly, our review shows that feature choice alone was insufficient as robustness depended on pairing features with denoising or noise-aware training. Classical filters, for example, Wiener and spectral subtraction, were common in infant cry studies [108], while ecology led the adoption of deep denoisers, augmentation strategies, and PCEN [3,21]. These practices align with broader advances in audio processing [10] but remain inconsistently applied. The insight here is that robustness is not feature-intrinsic but emerges from the integration of features, denoising, and evaluation metrics.

### Performance Outcomes

Reported accuracies clustered around 95%‐100%, creating the impression of ceiling-level performance. Yet, these results were largely driven by tier B pipelines tested in clean conditions, especially in infant cry datasets [47,109]. Tier A studies, particularly in ecological monitoring, reported more variable accuracies (approximately 75%‐95%) because they were evaluated under realistic noise conditions [64,104]. This variance is not a weakness but evidence of genuine robustness testing. It highlights the risk of publication bias: inflated best-case results dominate the literature, while average-case resilience is underreported. Interpreting these outcomes, therefore, requires caution. The broader implication is that progress in bioacoustics cannot be judged by peak accuracy alone, but by the consistency of performance under noise.

### Deployment and Translation

Deployment was reported in only 14.3% (19/132) of studies, nearly all from tier A pipelines. Infant cry applications emphasized mobile apps and caregiver support tools [110,111], prioritizing interpretability and immediacy but facing constraints around data privacy and ethics. Ecological deployments leveraged IoT networks, sensors, and open-source platforms to enable scalable biodiversity monitoring [61,98,112]. Bioacoustics surveillance deployments were rare. The absence of tier B baselines in deployment confirms that robustness is a prerequisite for translation. Domain-specific contrasts are clear: neonatal pipelines must prioritize ethical safeguards and clinician trust, while ecological pipelines require scalability, automation, and energy efficiency.

### Limitations and Future Direction

Limited standardized, high-quality datasets in both clinical and ecological domains restricted comparability. Although we included non-English studies, reliance on automated translation may have introduced subtle interpretive inaccuracies, though independent reviewer checks mitigated this risk. The inclusion of these non-English studies did not change the direction of findings, as their reported outcomes were consistent with the broader evidence base.

To advance sustainable integration, bioacoustics research should (1) standardize evaluation by adopting shared benchmarks, harmonized SNR protocols, and open datasets across taxa and infant populations [13,14]; (2) strengthen robustness methods, extending lightweight denoisers, augmentation strategies, and federated learning to support real-world generalization [77]; (3) tailor deployment strategies, interpretability, and privacy-preserving approaches for neonatal monitoring [78,113]; and (4) foster cross-domain transfer: ecological augmentation strategies can inform infant cry robustness, while clinical interpretability standards can guide ecological applications [64,98].

The translational relevance of these findings extends beyond research. In clinical contexts, robust infant cry classification could support early diagnostics and caregiver decision-making. In ecology, noise-resilient monitoring systems can enhance biodiversity surveillance and conservation policy [98,112]. Future studies should explicitly bridge domains, evaluating not only technical performance but also usability, interpretability, and sustainability in deployment.

### Conclusions

This review demonstrates that progress in bioacoustics classification is shaped less by the abundance of models than by the rigor of robustness evaluation. Tier A pipelines that incorporated explicit denoising and resilience testing provided the most credible evidence of real-world applicability, while tier B baselines, though often reporting high accuracies, rarely translated into deployment. Domain-specific contrasts further underscore that infant cry pipelines must prioritize interpretability and privacy, whereas ecological systems require scalable, energy-efficient designs.

Looking ahead, 3 levels of priority emerge. Immediate priorities include the creation of standardized, noise-augmented benchmark datasets and consistent reporting of preprocessing and denoising protocols. Short-term goals involve systematic evaluation of feature-model pairings across infant cry and ecological applications, coupled with pilot deployment studies in neonatal and field monitoring settings. Longer-term priorities focus on scaling deployment through cross-domain generalization methods (eg, transfer learning and federated learning), the development of lightweight edge-ready models, and the integration of interpretability and privacy safeguards for sustainable adoption.

By integrating insights on feature extraction, denoising, model architectures, and deployment, this review advances a cross-domain understanding of noise-resilient bioacoustics and provides a roadmap for future research. Moving beyond peak accuracies toward consistent robustness across diverse acoustic conditions will be key to translating methodological advances into reliable digital health and biodiversity conservation tools, with noise resilience as the cornerstone of sustainable impact.

## Supplementary material

10.2196/80089 Multimedia Appendix 1 PICO framework of the research questions. PICO: population, intervention, comparison, outcome.

10.2196/80089 Multimedia Appendix 2 Databases and search terms used in the search and selection of reviewed studies.

10.2196/80089 Multimedia Appendix 3 Data synthesis.

10.2196/80089 Multimedia Appendix 4 Databases and search terms used in the literature search.

10.2196/80089 Checklist 1 PRISMA checklist.

10.2196/80089 Checklist 2 TRIPOD checklist.

## Abbreviations

AUC: area under the receiver operating characteristic curve
CNN: convolutional neural network
CRNN: convolutional recurrent neural network
GAN: generative adversarial network
IoT: Internet of Things
LPCC: linear predictive cepstral coefficient
MECIR: Methodological Expectations of Cochrane Intervention Reviews
MFCC: Mel frequency cepstral coefficient
NICU: neonatal intensive care unit
OSF: Open Science Framework
PCEN: per-channel energy normalization
PICO: population, intervention, comparison, outcome
PRISMA: Preferred Reporting Items for Systematic Reviews and Meta-Analyses
RNN: recurrent neural network
RQ: review question
SNR: signal-to-noise ratio
SVM: support vector machine
TRIPOD: Transparent Reporting of a Multivariable Prediction Model for Individual Prognosis or Diagnosis
