# Supplementary material for: Noise-Resilient Bioacoustics Feature Extraction Methods and Their Implications on Audio Classification Performance: Systematic Review
Source: JMIR Biomed Eng. 2025 Dec 16;10:e80089. doi: 10.2196/80089 (PMC12707801; doi:10.2196/80089)
Supplement: Multimedia Appendix 2 [file biomedeng-v10-e80089-s002.docx]

**Databases and search terms used in the search and selection of reviewed studies**

| **Database** | **Search Terms and Queries** | **Records**  **Retrieved** |
| --- | --- | --- |
| Google Scholar | "bioacoustics" OR "infant cry classification" and "animal vocalization recognition" AND "feature extraction" AND ("MFCC" OR "spectrogram" OR "wavelet") AND  "Classification model" AND ("denoising" OR "noise robust" OR "signal enhancement") | 3098 |
|  | Filter was set to exclude all English and provide studies in all other languages | 246 |
| IEEE Xplore | (("bioacoustics" OR "infant cry" OR "animal vocalizations" OR "acoustic signal") AND  ("feature extraction" OR "MFCC" OR "spectrogram" OR "wavelet transform" OR "filterbank" OR "cepstral features") AND  ("classification" OR "machine learning" OR "deep learning" OR "CNN" OR "RNN" OR "neural networks") AND  ("denoising" OR "noise resilience" OR "noise robustness" OR "signal enhancement")) | 826 |
| ScienceDirect | TITLE-ABSTR-KEY (("bioacoustics" OR "infant cry classification" OR "animal call recognition" OR "bioacoustic signal") AND  ("feature extraction" OR "spectrogram" OR "MFCC" OR "wavelet packet" OR "filterbank" OR "Teager energy") AND  ("classification models" OR "machine learning" OR "deep learning" OR "neural networks") AND  ("denoising" OR "noise suppression" OR "noise robust")) | 503 |
| Scopus | TITLE-ABS-KEY (("bioacoustics" OR "animal calls" OR "infant cries") AND  ("feature extraction" OR "spectrogram" OR "MFCC" OR "filterbank" OR "wavelet transform" OR "cepstral coefficients") AND  ("classification" OR "machine learning" OR "deep learning" OR "neural networks") AND  ("noise-robust" OR "denoising" OR "noise-aware training" OR "signal enhancement")) | 363 |
| Web of Science | TS=("bioacoustics" OR "infant cry" OR "animal vocalizations") AND  TS=("feature extraction" OR "cepstral features" OR "spectrogram" OR "MFCC" OR "wavelet transform" OR "filterbank") AND  TS=("classification" OR "machine learning" OR "deep learning" OR "neural network") AND  TS=("denoising" OR "noise suppression" OR "noise robustness" OR "noise-aware training") | 206 |
| ACM Library | Abstract:("bioacoustics" OR "infant cry" OR "animal sound classification") AND  Abstract:("feature extraction" OR "MFCC" OR "spectrogram" OR "filterbank" OR "wavelet") AND  Abstract:("classification" OR "CNN" OR "RNN" OR "neural network") AND  Abstract:("denoising" OR "noise resilience" OR "signal enhancement") | 134 |
| SciELO | (bioacústica OR ecoacústica)  AND (clasificación OR reconhecimento OR classificação)  AND (ruido OR ruidoso OR “resiliente al ruido” OR “resistente ao ruído”)  AND (MFCC OR “coeficientes cepstrais” OR “coeficientes cepstrales”)  AND (“máquina de vectores de soporte” OR SVM OR “rede neural convolucional” OR CNN) which translates to (bioacoustics OR ecoacoustics) AND (classification OR recognition OR categorization) AND (noise OR noisy OR “noise-resilient” OR “noise-resistant”) AND (MFCC OR “cepstral coefficients”) AND (“support vector machine” OR SVM OR “convolutional neural network” OR CNN) | 58 |
| CNKI | ("生物声学" OR "生态声学")  AND (分类 OR 识别 OR 检测)  AND (噪声 OR 抗噪 OR 干扰)  AND (MFCC OR 倒谱系数)  AND (SVM OR 支持向量机 OR CNN OR 卷积神经网络) which translates to ("bioacoustics" OR "ecoacoustics") AND ("classification" OR "recognition" OR "detection") AND ("noise" OR "noise-resistant" OR "interference") AND (MFCC OR "cepstral coefficients") AND (SVM OR "support vector machine" OR CNN OR "convolutional neural network") | 28 |
| **Total Records Retrieved** | | 5462 |
